# The Dayside Ionosphere of Mars as Controlled by the Interplay Between Solar Wind Dynamic Pressure and Crustal Magnetic Field Strength

**DOI:** 10.1029/2024GL110838

**Published:** 2024-11-22

**Authors:** JunFeng Qin, Shannon Curry, Dave Mitchell, Shaosui Xu, Robert Lillis, Laila Andersson

**Affiliations:** ^1^ Space Sciences Laboratory University of California Berkeley CA USA; ^2^ Laboratory for Atmospheric and Space Physics University of Colorado Boulder CO USA; ^3^ Department of Astrophysical & Planetary Sciences University of Colorado Boulder CO USA

**Keywords:** Martian upper ionosphere, crustal magnetic field, solar wind dynamic pressure, electron density, magnetic topology

## Abstract

We investigate how the Martian dayside ionospheric structure is modified by crustal magnetic field (CMF) strength and upstream solar wind pressure by analyzing electron density data from the Langmuir Probe and Waves instrument onboard the MAVEN (Mars Atmosphere and Volatile EvolutioN) spacecraft. We find that the electron density above the exobase is anticorrelated with the ratio of solar wind's normal dynamic pressure ($P_{\text{SW}⊥}$) to CMF magnetic pressure ($P_{\text{CMF}}$). We also analyze the electron density behavior across different magnetic topologies as a function of $P_{\text{SW}⊥}/P_{\text{CMF}}$. The extremely low electron density in the draped topology relates to ionopause‐like structures. The lower electron density in the closed and open topology under higher $P_{\text{SW}⊥}/P_{\text{CMF}}$ may be attributed to a downward force, potentially the *J* × *B* force in the case of closed topology. This study highlights the complex interplay between solar wind and CMF in influencing the Martian dayside upper ionosphere.

## Key Points

- The electron density in the Martian dayside upper ionosphere is anticorrelated with pressure ratio of solar wind to crustal magnetic field
- The electron density in closed, open, and draped topology behaves differently as a function of this ratio
- The *J* × *B* force may play an important role in the effect of crustal magnetic field and solar wind conditions on the Martian upper ionosphere

## 1 Introduction

Present day Mars lacks a global magnetic field that can decelerate and deflect the incoming solar wind. As a result, the solar wind interacts directly with the upper atmosphere. The Martian ionosphere, due to its high conductivity, subsequently plays the role of deflecting the solar wind (Luhmann et al., 1991). Currents are generated in the ionosphere and magnetosphere of Mars, and through those currents, an induced magnetic field is built up that decelerates and deflects the shocked solar wind (Akalin et al., 2010; Halekas et al., 2021; Ramstad et al., 2020). On the dayside, the induced magnetic field generally takes the direction of the Interplanetary Magnetic Field (IMF) and “drapes” around the planet, so it is also called the “draped magnetic field” (Brain et al., 2006; Crider et al., 2004; Dubinin et al., 2021).

Due to the existence of the Martian crustal magnetic fields (CMF), the actual magnetic field on the Martian dayside deviates from the above described ideal draping geometry. Below the aforementioned “draped” topology, there are two other magnetic field topologies appearing with CMF: the “closed” and the “open.” The “closed” topology represents the closed magnetic loops connected at both ends to the lower ionosphere. The “open” topology represents magnetic field lines with one end connected to solar wind or sheath and another connected to the lower ionosphere (Brain et al., 2007; Vasyliunas, 1975; Xu et al., 2017). Those different types of topology can affect how and whether the plasma from lower ionosphere (below the exobase, i.e., about 180–200 km altitudes (Fowler et al., 2017; Gramapurohit & Rao, 2023)) can be transported to the upper ionosphere along the magnetic field lines. Because vertical plasma transport is the dominant process to shape the Martian upper ionosphere, the interaction between the CMF and induced magnetic field has a strong influence on the structure of the Martian ionosphere.

Previous observations have confirmed that the conditions of CMF and solar wind (which determines the strength and direction of the induced magnetic field) have obvious influences on the Martian upper ionosphere (above 200 km) (Andrews et al., 2015, 2023; Dubinin et al., 2016, 2019; Flynn et al., 2017; Fowler et al., 2019; Girazian et al., 2019; Qin et al., 2022; Ram et al., 2023; Withers et al., 2018). The upper ionosphere of Mars serves as the reservoir for atmospheric escape (e.g., photochemical escape and ion transportation) (Cravens et al., 2017; Lillis et al., 2017). Since atmospheric escape rates are directly related to the density and temperature of the ionospheric plasma, understanding how and why those parameters vary under different CMF and solar wind conditions is important. Despite numerous observational studies, the precise physics governing the influences of these two factors on the Martian upper ionosphere remains elusive. The variations and structures of different magnetic topologies at ionospheric altitudes is one key to understand the behaviors of Martian upper ionosphere. Several studies have focused on this aspect (Weber et al., 2019; M. Wang et al., 2022; Xu et al., 2023) and found that the draped topology (and the closed topology below it) appears at lower altitudes under higher solar wind dynamic pressure and weaker CMF. Specifically, Fowler et al. (2019) observed that very low electron density cases in the upper ionosphere occur when the draped topology intrudes into lower altitudes under high solar wind dynamic pressure. This suggests that part of the variations in the electron density of the Martian upper ionosphere could be explained by the altitude variations of the draped topology. However, since the closed topology is the dominant topology in the Martian dayside upper ionosphere (Weber et al., 2019), there are very few studies investigating how and why the electron density within the closed and open topology could be affected by different solar wind and CMF conditions.

In this research, we aim to statistically investigate the behaviors of the Martian upper ionosphere under different CMF and solar wind conditions in the context of magnetic topology with the help of the long‐term observations made by the MAVEN spacecraft. The results can shed light on our understanding of how and why the Martian upper ionosphere can be influenced by CMF and solar wind conditions. We only focus on the electron density in the dayside upper ionosphere. Solar wind dynamic pressure data, magnetic topology information, CMF model outputs are included in the analysis. Details about the data will be introduced in Section 2. The main results will be shown in Section 3. Section 4 will include discussions on the interpretation of observational results, and Section 5 is the conclusion.

## 2 Data and Instruments

For this study, we use electron density and temperature data measured by the Langmuir Probe and Waves (LPW) instrument onboard the MAVEN spacecraft from October 2014 to November 2022. LPW is designed to measure the thermal electron density and temperature in the Martian ionosphere and detect waves therein. Its two Langmuir probes can measure electron density in the range of 100–10^6^/cm^3^ and electron temperature in the range of 500–50000 K with the fastest time resolution of ∼4 s at the lowest altitudes (Andersson et al., 2015). Details on the derivation and uncertainties of these data can be found in Ergun, Andersson, Fowler, Thaller, and Yelle (2021) and Ergun, Andersson, Fowler, and Thaller (2021). We adopt the electron density and temperature data with quality flags greater than 50 to ensure accuracy.

Solar wind dynamic pressure is calculated through the upstream ion density and velocity measured by the Solar Wind Ion Analyzer (SWIA) (Halekas et al., 2015) onboard the MAVEN spacecraft and averaged over each orbit. Since MAVEN cannot simultaneously measure the upstream solar wind and ionosphere, the solar wind dynamic pressure during the LPW electron density measurements is estimated by linearly interpolating the dynamic pressure measured in adjacent orbits. We discard any electron density data collected more than 3 hr from a solar wind dynamic pressure measurement. Due to this limitation, any variations in solar wind dynamic pressure that occur within a three‐hour window cannot be accounted for in our analysis. In the meantime, CMF strength is calculated based on the CMF model by Langlais et al. (2019).

Finally, we use magnetic topology information provided by Xu et al. (2019) to infer the topology at the location where electron density was measured. The topology is based on the analysis of the suprathermal electron spectrum and pitch angle distribution measured by SWEA (Mitchell et al., 2016) onboard the MAVEN spacecraft. Only the topology information before 2020 is available during this study.

## 3 Results

At first, a gauge is needed to incorporate the influences of CMF strength and solar wind dynamic pressure on the Martian upper ionosphere so that the effects of these two factors can be analyzed simultaneously. We choose to use the ratio of the solar wind normal dynamic pressure ($P_{\text{SW}⊥}$) to the CMF magnetic pressure ($P_{\text{CMF}}$). There are primarily two rationales for employing this ratio (i.e., $P_{\text{SW}⊥}/P_{\text{CMF}}$). First, previous research has found anti‐correlation of solar wind dynamic pressure (Girazian et al., 2019) and positive correlation of CMF strength (Andrews et al., 2023) with the electron density in the Martian dayside upper ionosphere, making it logical to use their ratio as a metric to evaluate their combined effects. Second, as elaborated in Section 1, the magnetic topology, which is contingent upon the interaction between the induced magnetic field and CMF, serves as the key factor in shaping the Martian upper ionosphere. Previous research (Crider et al. (2003) and Cravens et al. (2017)) has shown that the strength of the dayside induced draping magnetic field highly depends on the normal component of the upstream solar wind dynamic pressure, thus making it reasonable to use $P_{\text{SW}⊥}/P_{\text{CMF}}$ as a metric for gauging the interaction between the induced magnetic field and CMF.

This ratio can be expressed as:

(1) $$P_{\text{SW}⊥}/P_{\text{CMF}}=P_{\text{SW}}\cdot \cos^{2}\left(\text{SZA}\right)/P_{\text{CMF}}$$

In the above equation, $P_{\text{CMF}}$ (the magnetic pressure of CMF) is given by ${\left|B\right|}_{\text{CMF}}^{2}/{2\mu }_{0}$ (${\left|B\right|}_{\text{CMF}}$ represents the strength of the CMF, and $\mu_{0}$ is the vacuum permeability) and can be calculated through the CMF model (see Section 2); $P_{\text{SW}}$ (the upstream solar wind dynamic pressure) can be derived through SWIA observations (see Section 2), and SZA represents Solar Zenith Angle.

**Figure 1:**
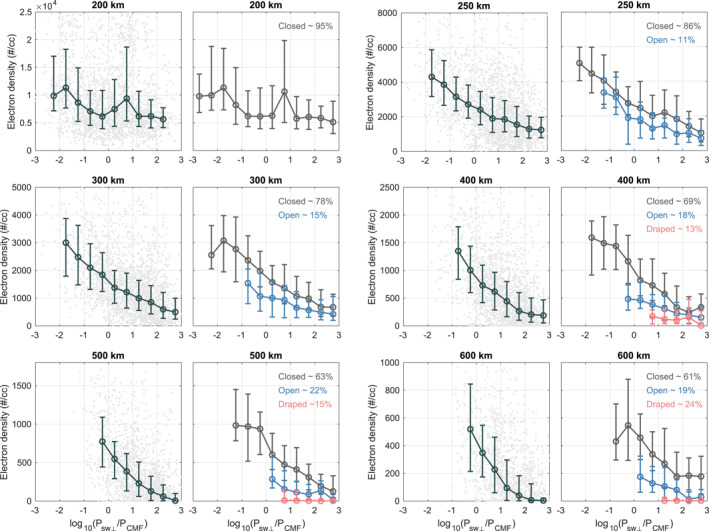
The dependence of electron density at 200, 250, 300, 400, 500, and 600 km (±5 km) on $P_{\text{SW}⊥}/P_{\text{CMF}}$. Subplots in columns 1 and 3 show the electron density data distribution (gray dots, only representing parts of data that are randomly selected) and their statistics (dark blue error bars, for the group with number of data points larger than 100). Subplots in columns 2 and 4 show the statistics for the electron density from different topology (dark gray for closed, blue for open, and red for draped, for the group with number of data points larger than 20) along with their occurrence in the upper right corner. The data points in each subplot are divided into 12 groups with a bin width of 0.5 according to different $\log_{10}\left(P_{\text{SW}⊥}/P_{\text{CMF}}\right)$ values. The error bars show the median value (circle) and upper/lower quantiles (horizontal bars) for each group of data. Note that the statistics for the electron density data from a specific topology with an occurrence lower than 10% at a specific altitude are not shown.

In Figure 1 we show how the dayside ionospheric electron density at six altitudes‐ 200, 250, 300, 400, 500, and 600 km (±5 km)‐ is correlated with $P_{\text{SW}⊥}/P_{\text{CMF}}$. Note that $P_{\text{SW}⊥}/P_{\text{CMF}}$ is calculated based on the exact location (latitude, longitude, altitude and SZA) and corresponding time of each electron density data. Latitude, longitude, and altitude determine ${\left|B\right|}_{\text{CMF}}$, whereas time determines $P_{\text{SW}}$. Only the electron density data with SZA lower than 60° are included to fulfill Equation 1 (Cravens et al., 2017; Crider et al., 2003). The distribution and statistics for all the electron density data are shown in columns 1 and 3, and the statistics for the electron density data from specific magnetic topology are shown in columns 2 and 4. As the topology information was only available up to Year 2019 at the time of this analysis, we only include the electron density data before Year 2020 in columns 2 and 4. We provide the distribution of the electron density data from different topologies as a function of $P_{\text{SW}⊥}/P_{\text{CMF}}$ in Figure S2 in Supporting Information S1.

Figure 1 demonstrates that the electron density in the Martian dayside upper ionosphere is anti‐correlated with the pressure ratio of solar wind to crustal magnetic fields. This is consistent with previous results (Andrews et al., 2023; Girazian et al., 2019; references in Section 1). From low $P_{\text{SW}⊥}/P_{\text{CMF}}$ (i.e., strong CMF and low solar wind dynamic pressure) to high $P_{\text{SW}⊥}/P_{\text{CMF}}$ (i.e., weak CMF and high solar wind dynamic pressure), the median values of dayside ionospheric electron density change from about fourfold (at 250 km) to over tenfold (above 500 km), so this ratio is clearly a key factor governing the Martian upper ionosphere.

When examining the behaviors of dayside electron density across different magnetic topologies as shown in columns 2 and 4 of Figure 1, the analysis becomes more intricate and intriguing. The draped topology (red) is mainly observed at higher altitudes (above 300 km) and tends to occur when $P_{\text{SW}⊥}$ exceeds $P_{\text{CMF}}$, and the electron density in the draped topology appears notably low. The closed topology (gray), as the dominant magnetic topology type in the Martian upper ionosphere, is generally distributed across the whole range of $P_{\text{SW}⊥}/P_{\text{CMF}}$, and the electron density in closed topology exhibits a negative correlation with $P_{\text{SW}⊥}/P_{\text{CMF}}$. As for the open topology (blue), the electron density in it mimics the anti‐correlation observed in the closed topology but with lower values. The median electron density in open topology is found notably lower than that in closed topology, with a ratio from approximately 0.8 at 250 km to below 0.5 at 600 km.

We also find that draped (and open) topology only accounts for a minor portion of all the magnetic topologies at upper ionospheric altitudes (less than 15% (%22) in our research and less than 20% (30%) in Weber et al. (2019) at the altitude of 200–500 km), while closed topology predominates in the Martian dayside upper ionosphere. The physical processes for the Martian upper ionosphere in the draped and open topology have been paid some attention (e.g., Fowler et al., 2019; Xu et al., 2020, 2023) while that in closed topology remains unclear. In the next section, we will analyze the behaviors of electron density in each magnetic topology. Elucidating those behaviors is the key to understanding how CMF and solar wind interact with each other to shape the Martian upper ionosphere.

## 4 Discussion

In the previous section, we examined the behaviors of electron density in the Martian dayside upper ionosphere under varying $P_{\text{SW}⊥}/P_{\text{CMF}}$ values, and showed how magnetic topology modifies those behaviors. Magnetic topology plays a very important role in controlling plasma transport and shaping the Martian upper ionosphere, so in this section, we investigate further how magnetic topology acts as a bridge linking electron density with $P_{\text{SW}⊥}/P_{\text{CMF}}$ values.

Since the electron density data from different topologies exhibits divergent behaviors, we propose three independent explanations for the behaviors of electron density data in draped, closed, and open topologies:

1. The draped topology appears when induced magnetic field dominates over CMF, and prevents access to thermal plasma from the lower ionosphere. This could explain why the draped topology usually exists when $P_{\text{SW}⊥}/P_{\text{CMF}}$ > 1 and accompanies very low electron density.
2. Under larger $P_{\text{SW}⊥}/P_{\text{CMF}}$, a downward *J* × *B* force could arise in the closed topology and inhibit plasma upward transport. This could explain the lower electron density in closed topology under larger $P_{\text{SW}⊥}/P_{\text{CMF}}$.
3. The open topology, like the closed topology, also experiences a $P_{\text{SW}⊥}/P_{\text{CMF}}$‐modulated downward force but suffers greater plasma loss. This may explain why the electron density in the open topology exhibits a similar anti‐correlation with $P_{\text{SW}⊥}/P_{\text{CMF}}$ as observed in the closed topology, but with lower values.

In the subsequent subsections, we will delve into each of these explanations, providing detailed analyses to elucidate the underlying mechanisms driving the observed behaviors.

### 4.1 Electron Density in the Draped Topology

In Figure 1, we find that the draped topology is mainly observed at higher altitudes (above 300 km) and tends to occur when $P_{\text{SW}⊥}$ exceeds $P_{\text{CMF}}$, and the electron density in the draped topology appears notably low.

As introduced in previous sections, the normal component of solar wind dynamic pressure, $P_{\text{SW}⊥}$, determines the magnetic pressure of dayside induced magnetic field. When $P_{\text{SW}⊥}>\;P_{\text{CMF}}$, the induced magnetic field dominates over CMF. The former mainly contributes to the draped part of magnetic field while the latter gives the closed and open parts of magnetic field. Given this premise, it's reasonable that the draped topology tends to appear when $P_{\text{SW}⊥}>\;P_{\text{CMF}}$. This scenario is consistent with previous findings that the draped topology intrudes to lower altitudes under higher solar wind dynamic pressure and weaker CMF (Garnier et al., 2017; Y. Wang et al., 2022; Xu et al., 2023).

Different types of magnetic topology can affect how and whether the plasma from lower ionosphere can be transported to the upper ionosphere (Fowler et al., 2017; Gramapurohit & Rao, 2023). In closed topology, both footpoints of the magnetic field line connect to the ionosphere below 200 km, so the thermal plasma from below 200 km can transport along closed loops to higher altitudes. However, in draped topology, both footpoints of the magnetic field line connect to the solar wind or magnetosheath, where the thermal plasma density is significantly low. Consequently, there will be very limited thermal plasma that can enter the draped topology, explaining why the electron density in draped topology is very low.

**Figure 2:**
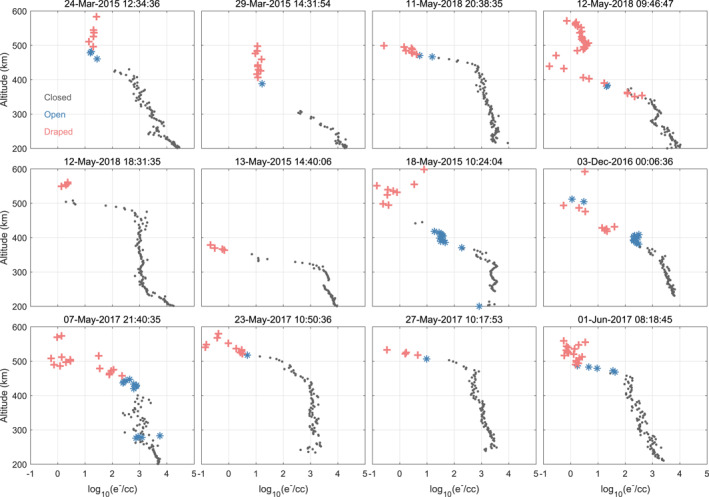
Electron density profiles color‐coded by different topology types. Red crosses represent electron density in draped topology; blue stars represent electron density in open topology; black dots represent electron density in closed topology. The first five subplots are inbound observations, and others are outbound observations.

In Figure 2 we show that the existence of draped topology can indeed lead to very low electron density. We plot 12 example profiles of electron density measured by LPW and color‐coded by different topology types (black dots for closed, blue stars for open, and red crosses for draped). In each of these cases, a transition from closed/open (lower altitudes) to draped topology (higher altitudes) can be found. Such a transition indicates that the MAVEN spacecraft is crossing the boundary between draped topology and closed/open topology. Notably, whenever such a transition occurs, the electron density data measured in the draped topology drops to very low values, showing that the very low electron density is strongly linked with the draped topology. Those drops of electron density can be called as ionopause‐like structures. For reference, we include an example electron density profile in Figure S3 in Supporting Information S1 that does not exhibit an ionopause‐like structure. Unlike the 12 profiles shown in Figure 2, in this case, the electron density decreases gradually with increasing altitude and remains above 100/cm^3^ at 600 km. We examined a total of 276 profiles captured entirely on the dayside (SZA < 60°) that include draped topology below 600 km, and found that approximately 80% (220 profiles) exhibit ionopause‐like structures at the transition altitude between the closed/open and draped topology. These profiles can be found in Qin (2024). The results of Xu et al. (2023) are also consistent with the above scenario. Their research reveals that large drops of planetary ion density occur across the Photo‐Electron Boundary, the topological boundary for closed/open and draped topology, indicating a connection between the very low electron density and the appearing of draped topology.

### 4.2 Electron Density in the Closed Topology

In Figure 1, we find that, as the dominant magnetic topology in the Martian upper ionosphere, the closed topology is generally distributed across the entire range of $P_{\text{SW}⊥}/P_{\text{CMF}}$, and the electron density in closed topology exhibits an anti‐correlation with $P_{\text{SW}⊥}/P_{\text{CMF}}$.

In the closed topology, plasma from lower ionosphere can freely transport into upper ionosphere along closed magnetic field lines (i.e., closed loops). A steady‐state assumption for the MHD (magnetohydrodynamic) equation of motion can be applied to the magnetized plasma in the closed topology (check Text S3 in Supporting Information S1):

(2) $$-\vec{\nabla }P_{i}-\vec{\nabla }P_{e}+n_{i}m_{i}\vec{g}+\vec{f}+J\times B=0$$

In the above equation, $P_{i,e}$ represents the thermal pressure of ions/electrons, $n_{i}$ represents ion density, $m_{i}$ denotes ion mass, $\vec{g}$ is the gravitational acceleration (∼3.7 m/s^2^ on Mars and assumed to be constant across different altitudes), $\vec{f}$ represents collisions between plasma and neutral atmosphere, and J is the current in the upper ionosphere.

The J×B term in Equation 2 is likely the primary factor responsible for the anti‐correlation between the electron density in the closed topology and $P_{\text{SW}⊥}/P_{\text{CMF}}$: the term $\vec{f}$ is unlikely to be relevant to $P_{\text{SW}⊥}/P_{\text{CMF}}$ and should be considered negligible at the altitudes of upper ionosphere (i.e., above the exobase) (Hanley et al., 2021; Schunk & Nagy, 2009), while J is highly related to CMF and solar wind conditions (Halekas et al., 2017; Ramstad et al., 2020). Previous research also indicates that J×B force is significant in the Martian ionosphere (Halekas et al., 2017).

Here we analyze how this J×B term affects the electron density in the closed topology under different $P_{\text{SW}⊥}/P_{\text{CMF}}$ values. We focus on the vertical component of Equation 2. By assuming the temperature of ions is half of that of electrons (Hanley et al., 2021; Matta et al., 2014), we get:

(3) $${\left(-\vec{\nabla }P_{i}-\vec{\nabla }P_{e}\right)}_{⊥}=-\frac{\partial }{\partial h}\left(n_{i}k_{B}T_{i}+n_{e}{k_{B}T}_{e}\right)=-\frac{3}{2}\frac{\partial }{\partial h}\left(n_{e}{k_{B}T}_{e}\right)$$

Considering that $O_{2}^{+}$ is the dominant ions at 200–400 km, $m_{i}$ equals 32 Da, so:

(4) $${\left(n_{i}m_{i}\vec{g}\right)}_{⊥}=-32n_{e}m_{u}g$$

In the above equations, the symbol ⊥ represents the vertical component, h represents altitude, $k_{B}$ is Boltzmann constant, and $m_{u}$ is unified atomic mass.

**Figure 3:**
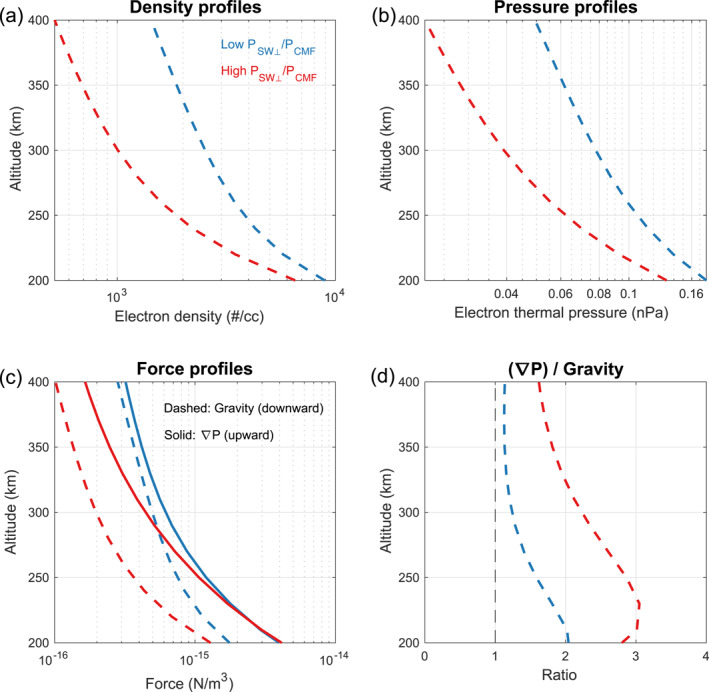
Profiles of median electron density (panel a), median electron thermal pressure (panel b), and forces for the Martian upper ionosphere at 200–400 km under low $P_{\text{SW}⊥}/P_{\text{CMF}}$ condition (blue) and high $P_{\text{SW}⊥}/P_{\text{CMF}}$ condition (red) for the closed topology. Panel d shows the ratio of pressure gradient to the gravitational force. Details about how the profiles in panels (a) and (b) are derived can be found in Text S4 in Supporting Information S1.

With the above equations, the vertical components of pressure gradient ($-\vec{\nabla }P_{i}-\vec{\nabla }P_{e}$) and gravitational force ($n_{i}m_{i}\vec{g}$) can be calculated based on the values of $n_{e}$ and $T_{e}$ observed by LPW (see Section 2). In panels a and b of Figure 3, we show the profiles of $n_{e}$ (panel a) and profiles of $n_{e}{k_{B}T}_{e}$ (panel b) under two conditions: $P_{\text{SW}⊥}/P_{\text{CMF}}$ < 0.3 (blue) and $P_{\text{SW}⊥}/P_{\text{CMF}}$ > 3 (red). These profiles are derived by fitting the median values of observations at different altitudes within the dayside (SZA < 60°) closed topology (see Text S4 in Supporting Information S1). In panel c, we show the profiles of ${\left(n_{i}m_{i}\vec{g}\right)}_{⊥}$ and ${\left(-\vec{\nabla }P_{i}-\vec{\nabla }P_{e}\right)}_{⊥}$ calculated through Equations 3 and 4. Note that the pressure gradient is upward, while the gravitational force is downward. Panel d of Figure 3 displays the absolute ratios between the pressure gradient and gravitational force.

To interpret the results shown in Figure 3, we revisit Equation 2. The collision between ions and neutrals is mainly significant below the exobase (∼200 km) (Hanley et al., 2021; Schunk & Nagy, 2009). Considering that the neutral density decreases exponentially with altitude, it's reasonable to assume that the friction force in Equation 2 can be neglected above 300 km. Then, there are only three forces in balance at these altitudes: the upward pressure gradient, the downward gravitational force, and the J×B force. Based on the results of Figure 3, the upward pressure gradient nearly matches the downward gravitational force under low $P_{\text{SW}⊥}/P_{\text{CMF}}$, but significantly larger than it under high $P_{\text{SW}⊥}/P_{\text{CMF}}$. This suggests that as $P_{\text{SW}⊥}/P_{\text{CMF}}$ increases, a downward J×B force should arise to account for this imbalance between the upward pressure gradient and the downward gravitational force. In this case, the absolute ratio between the upward pressure gradient and the downward gravitational force above 300 km ranges from 1.6 to 2.3 (see panel d of Figure 3), indicating that this downward *J* × *B* force is likely comparable in magnitude to the gravitational force.

From the view of MHD equation of motion (i.e., Equation 2), this downward force changes the thermal structure of the plasma in closed loops, leading to a smaller scale height of electron density. From the view of plasma transport, this downward force inhibits the upward transport of plasma from lower ionosphere. Both explanations can expect lower electron density under higher $P_{\text{SW}⊥}/P_{\text{CMF}}$, consistent with the results of Figure 1.

### 4.3 Electron Density in the Open Topology

In Figure 1, we find that the electron density in open topology mimics the trend of electron density in closed topology but with lower median values.

**Figure 4:**
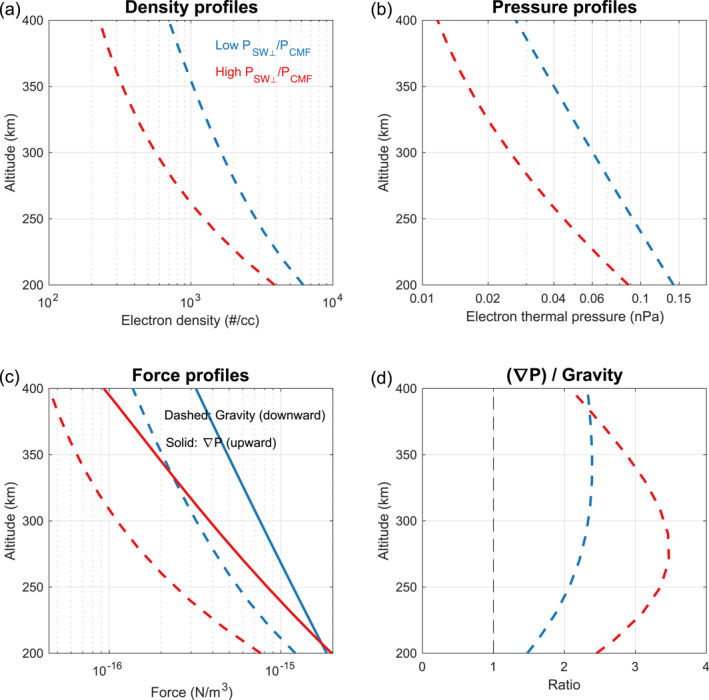
The same as Figure 3 but for the open topology. Details about how the profiles in panels (a) and (b) are derived can be found in Text S4 in Supporting Information S1.

Like closed loops, the plasma in the open topology is also governed by vertical plasma transport along the magnetic field lines. A steady‐state assumption for the MHD equation of motion can be applied here as well (check Text S3 in Supporting Information S1). Therefore, we use the same method as in Section 4.2 to analyze the vertical force balance in the open topology, with the results presented in Figure 4 in the same form as Figure 3.

In Figure 4d, a higher ratio of upward pressure gradient to the downward gravitational force is found under higher $P_{\text{SW}⊥}/P_{\text{CMF}}$ (red); even under low $P_{\text{SW}⊥}/P_{\text{CMF}}$ (blue), the upward pressure gradient is found more than twice of the downward gravitational force rather than in balance as in the case of closed topology (check Figure 3d). Similar to the analysis in Section 4.2, this implies the existence of a downward force term which increase as $P_{\text{SW}⊥}/P_{\text{CMF}}$ increases just like the case of closed topology but remains significant even under low $P_{\text{SW}⊥}/P_{\text{CMF}}$. This scenario could explain why the electron density in the open topology exhibits a similar anti‐correlation with $P_{\text{SW}⊥}/P_{\text{CMF}}$ as observed in the closed topology but with lower values.

It seems unlikely that this downward force term could be *J* × *B* as in the case of closed topology, considering that the magnetic field lines in the open topology are usually radial, making it difficult to induce a significant *J* × *B* term in the downward direction. One possibility is that suprathermal plasma from the solar wind, which has a low flux in the closed topology but significant in the open topology (Xu et al., 2015, 2019), may affect the force balance of the thermal plasma in the open topology. Since LPW can only observe thermal plasma (Andersson et al., 2015), this potential effect by suprathermal plasma is not included in our analysis. Solar Wind Electron Analyzer (SWEA) and SWIA onboard MAVEN are capable of observing suprathermal plasma, enabling further analysis based on these data sets.

Another difference between the closed and open topology lies in the plasma loss term: the plasma in the open topology can get lost directly to space while the plasma in the closed topology cannot. Accelerated planetary ion outflow has been found above open‐topology‐dominant magnetic cusp regions by many studies (Dubinin et al., 2009; Halekas et al., 2008; Lundin et al., 2006; Xu et al., 2020). This plasma outflow in the open topology likely results in lower electron density, also potentially explaining why the electron density from open topology is slightly lower than that from closed topology.

It should be noted that our MHD approach in Section 4.2 and 4.3 is quite simple and based solely on statistical results. The inertial term in the case of open topology may be significant under extreme conditions (see Text S3 in Supporting Information S1), and the amount and accuracy of LPW data would affect the results especially in the open topology case (see Text S4 in Supporting Information S1). However, this is the extent of what can be achieved in an observation‐based study. In the research based on pure MHD simulations, such as M. Wang et al. (2022), the force balance in the Martian upper ionosphere under different solar wind and CMF conditions could be examined in a more rigorous manner.

### 4.4 Comparison With Previous Work

The effects of solar wind dynamic pressure and CMF strength on electron density in the Martian dayside upper ionosphere, as shown in Figure 1, have been reported in many previous studies (see references in the Introduction) with similar results. In our research, we introduce two key innovations: (a) a new index, $P_{\text{SW}⊥}/P_{\text{CMF}}$, which combines the effects of both CMF strength and solar wind dynamic pressure and (b) an independent analysis of electron density behaviors under different solar wind and CMF conditions across closed, open, and draped topology. These aspects have not been addressed in previous research and proved crucial for advancing our understanding of how solar wind and CMF conditions affect the upper ionosphere.

Our interpretation of electron density behavior in the draped topology (Section 4.1) is consistent with the findings of Fowler et al. (2019). Specifically, we conduct a survey on ionopause‐like structures in the Martian upper ionosphere, showing that such structures appear in 80% of the transitions from closed/open to draped topology. Recent simulations by M. Wang et al. (2022) suggest that the altitudes of MPB (magnetic pileup boundary, i.e., the bottom of the draped topology) are influenced not only by solar wind dynamic pressure and CMF strength but also by IMF direction, which should be considered in future observational studies.

The behaviors of electron density in the closed and open topology (Figure 1), along with our interpretations based on an MHD approach (Section 4.2 and 4.3), has not been addressed in previous observational research. The closed topology is the dominant topology in the Martian upper ionosphere, while the open topology is crucial for understanding cold ion escape (Dong et al., 2024). Therefore, understanding the behavior of upper ionosphere in closed and open topology under different solar wind and CMF conditions is essential. Our results in Figures 1, 3, and 4 provide quantitative comparisons between plasma in closed and open topology, which potentially can be used to calculate escape rate and make comparisons with simulation results in future work.

## 5 Conclusion

In this research, we investigate the effects of CMF strength and solar wind dynamic pressures on the Martian upper ionosphere. Our findings reveal a distinct anti‐correlation between the electron density of the Martian dayside upper ionosphere and the ratio of the solar wind's normal dynamic pressure to the CMF pressure ($P_{\text{SW}⊥}/P_{\text{CMF}}$). The electron density from draped, closed, and open topology yields different behaviors as a function of $P_{\text{SW}⊥}/P_{\text{CMF}}$. The draped topology occurs when $P_{\text{SW}⊥}/P_{\text{CMF}}$ > 1, and causes very low electron density due to limited access to the plasma from lower ionosphere. High $P_{\text{SW}⊥}/P_{\text{CMF}}$ can lead to a downward J×B force in the closed topology, inhibiting plasma upward transportation and leading to low electron density. The open topology experiences a similar $P_{\text{SW}⊥}/P_{\text{CMF}}$‐modulated downward force but suffers greater plasma loss, yielding lower electron density than that in closed topology. These results enhance our understanding of Martian upper ionosphere, and can shed light on how the Martian CMF, a unique planetary feature in the solar system, interacts with solar wind and affects the Martian ionosphere.

## Supporting information

Supporting Information S1

## Data Availability

The solar wind dynamic pressure data can be accessed at https://homepage.physics.uiowa.edu/~jhalekas/drivers.html. Electron density data (Andersson, 2024a), electron temperature data (Andersson, 2024b), and in‐situ magnetic field data (Connerney, 2024) are available through the Planetary Data System. Crustal magnetic field model of Langlais et al. (2019) and magnetic field topology information can be generated using MAVEN IDL Toolkit (https://lasp.colorado.edu/maven/sdc/public/pages/software.html). Supplementary documents of 276 profiles showing the correlation between ionopause‐like structures and draped topology can be found at Qin (2024).
