# Supplementary material for: The Dayside Ionosphere of Mars as Controlled by the Interplay Between Solar Wind Dynamic Pressure and Crustal Magnetic Field Strength
Source: Geophys Res Lett. 2024 Nov 22;51(22):e2024GL110838. doi: 10.1029/2024GL110838 (PMC11583551; doi:10.1029/2024GL110838)
Supplement: Supplementary file 1 — Supporting Information S1 [file GRL-51-0-s001.pdf]

*[Geophysical Research Letter]*

Supporting Information for

# [The Dayside Ionosphere of Mars as controlled by the Interplay Between Solar Wind Dynamic Pressure and CMF Strength]

**JunFeng Qin[1*], Shannon Curry[2,3], Dave Mitchell[1], Shaosui Xu[1], Robert Lillis[1], Laila Andersson[2]**

[1]Space Sciences Laboratory, University of California, Berkeley, CA, USA.

[2]Laboratory for Atmospheric and Space Physics, University of Colorado, Boulder, CO, USA.

[3]Department of Astrophysical & Planetary Sciences, University of Colorado, Boulder, CO, USA.

Corresponding author: JunFeng Qin (jfqin@berkeley.edu)

## Contents of this file

Texts S1, S2, S3, and S4
Figures S1, S2, S3, S4, S5, and S6

**Text S1: comparison between LPW and SWEA measured electron density of the Martian upper ionosphere.**

According to Andersson et al. [2015], the Langmuir Probe and Waves (LPW) instrument onboard the MAVEN spacecraft can get trustable measurements on the electron density in the range of 100-$10^6$ /$cm^3$. Readers may notice that in Figure 1 and 2 there are parts of electron density data lower than 100/$cm^3$. Those electron density data are mainly from the draped topology, where the dominant electrons are super-thermal electrons. The density of super-thermal electrons can be measured by the Solar Wind Electron Analyzer (SWEA) instrument [Mitchell et al., 2016] onboard MAVEN, so a comparison between the results of LPW and SWEA in the draped topology can be made to check the credibility of LPW's results that are lower than 100/$cm^3$.

Figure S1 shows the electron density measurements of LPW and SWEA in one case that MAVEN crossed the draped topology twice. The two crossings of draped topology are marked by dashed blue lines (300-340 km, and above 500 km). We can find that in the draped topology, the results of LPW, although lower than 100/$cm^3$, are quite consistent with the results of SWEA. Outside of the draped topology (i.e., closed or open), the thermal electrons become the dominant part, so the results of LPW get far larger than the results of SWEA.

Based on the results of Figure 1S, we can conclude that, although the measurements of LPW lower than 100/$cm^3$ may not be as accurate as those higher than 100/$cm^3$, they should be at least trustable on the order of magnitude. So the electron density data points lower than 100/$cm^3$ would not influence the main results of Figures 1 and 2.

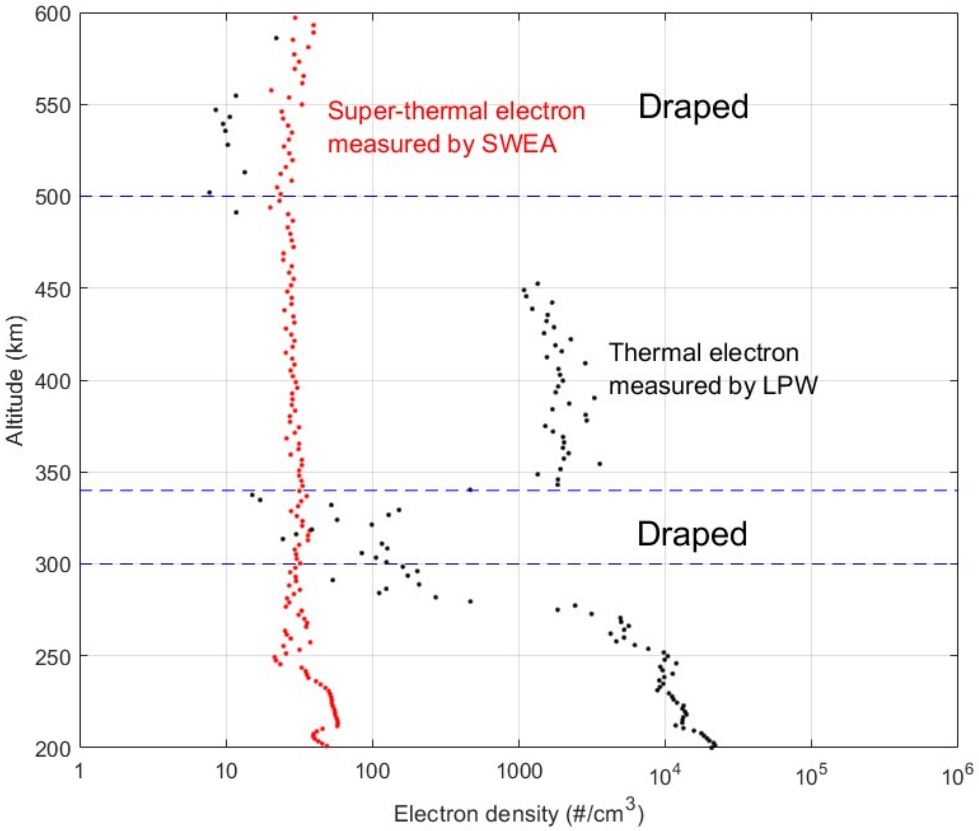


**Figure S1.** Measurements on the electron density made by SWE (red) and LPW (black) during 02:13-02:22 on 25 March 2015.

## Text S2: data distribution in Figure 1

In Figure 1 we only show the statistics for the electron density from different topologies as a function of $P_{SW\perp}/P_{CMF}$. Here we show the distribution of those electron density data.

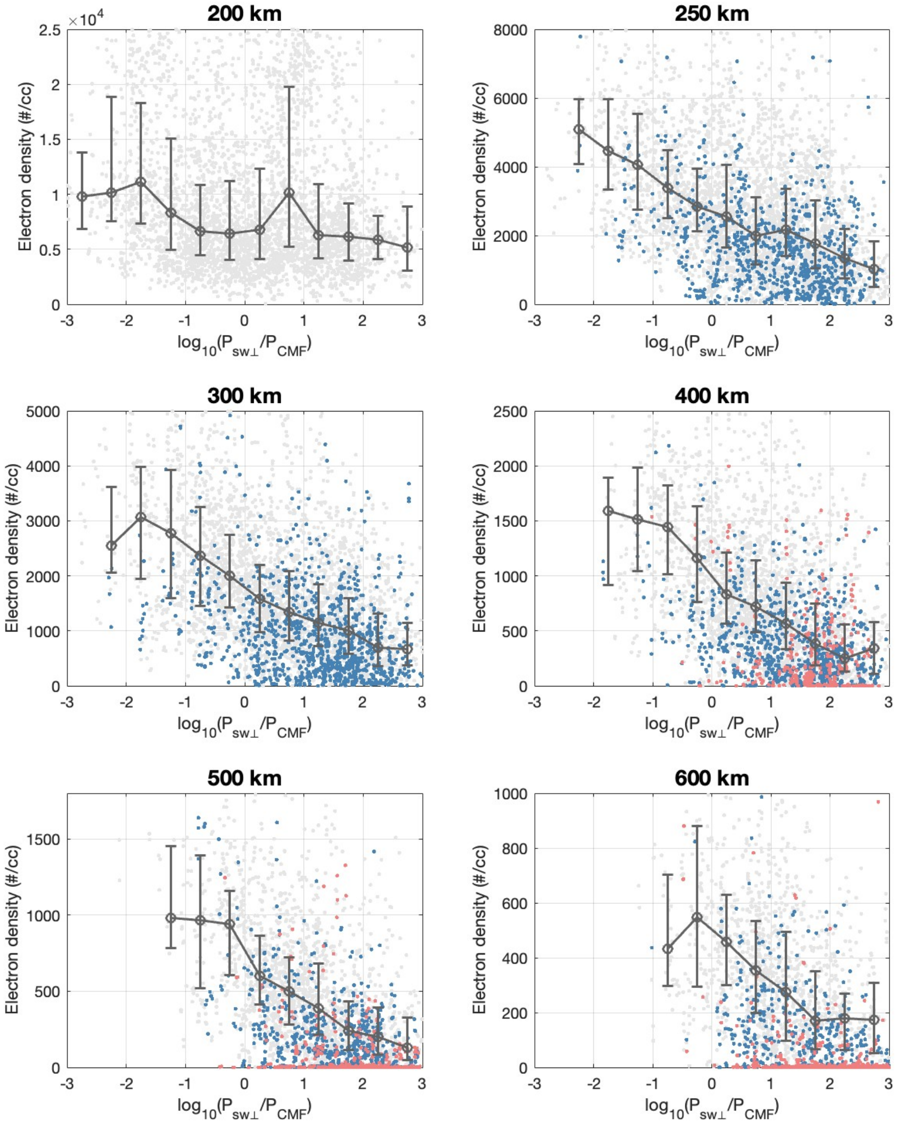


**Figure S2.** The dependence of electron density from different types of magnetic topology on the ratio $\boldsymbol{P_{SW\perp}/P_{CMF}}$ at different altitudes. The grey dots represent electron density data points from closed topology. The blue dots represent electron density data points from open topology. The red dots represent electron density data points from draped topology. Black error bars show the statistics for the electron density from closed topology (same as Figure 1).

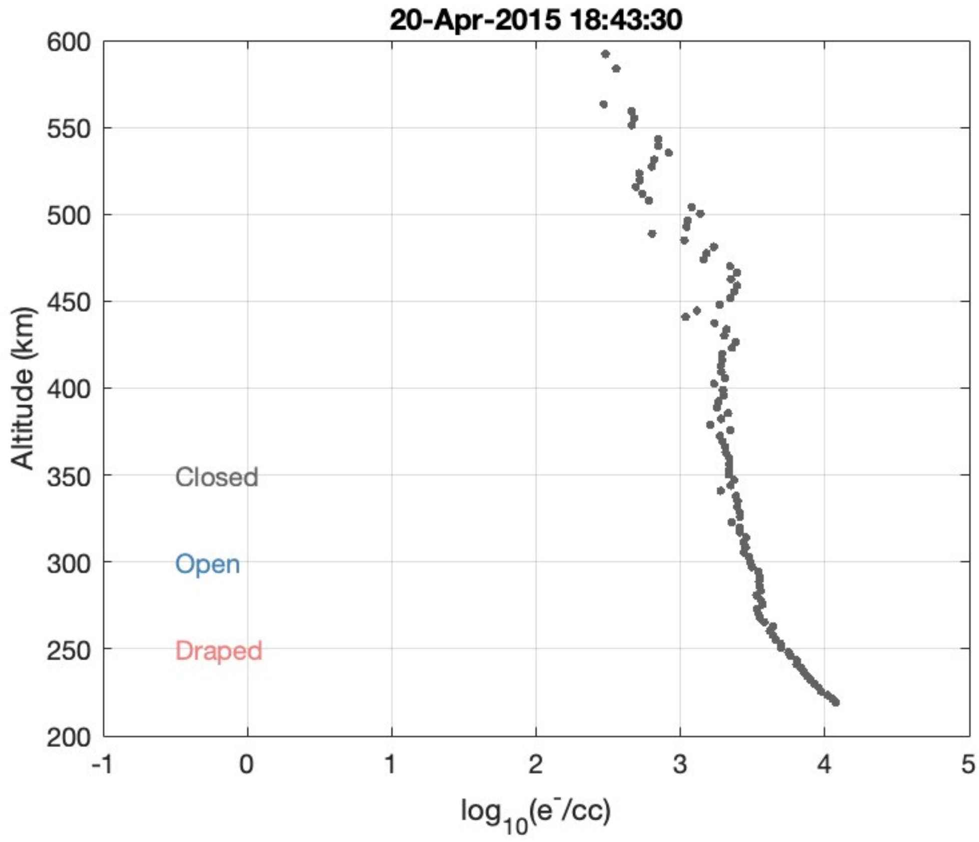


**Figure S3.** One electron density profile observed by LPW in which there is no draped topology and no ionopause-like structure.

**Text S3: evaluation of the inertial term in the MHD equation of motion for the Martian dayside upper ionosphere conditions.**

The full form of MHD motion equation is:

$$n_i m_i \frac{d\vec{V}_i}{dt} = n_i m_i [\frac{\partial \vec{V}_i}{\partial t} + (\vec{V}_i \cdot \nabla)\vec{V}_i] = -\vec{\nabla} P_i - \vec{\nabla} P_e + n_i m_i \vec{g} + \vec{f} + J \times B$$

In equation (2) we omitted the inertial term(s) on the left side and assumed a steady-state condition. Here, we evaluate the magnitudes of the inertial term(s) relative to the gravitational force on the right side to justify why a steady-state condition can be adopted for the closed and open topology in the Martian dayside upper ionosphere. Note that we only focus on the vertical component of the above equation and the equation (2).

Figure S3 shows the results of an MHD (magnetohydrodynamic) simulation [Ma et al., 2014] on the $O_2^+$ vertical velocity in the Martian upper ionosphere.

For the closed topology, under different crustal magnetic field conditions, and at altitudes between 300-400 km, the typical velocity for $O_2^+$ ($|\vec{V}_i|$) is ~ 100 m/s, with a typical velocity variation ($|d\vec{V}_i|$) of ~ 100 m/s. The horizontal span ($H$) is ~ 100 km (from 300 km to 400 km), thus the typical time span ($T$) is:

$$T \approx \frac{H}{|\vec{V}_i|} = \frac{100\,km}{100\,m/s} = 1000\,s$$

Then we have:

$$\frac{d\vec{V}_i}{dt} \approx \frac{|d\vec{V}_i|}{T} = \frac{100\,m/s}{1000\,s} = 0.1\,m/s^2$$

Which is over one order of magnitude smaller than the gravitational term considering $\vec{g}$ is ~3.7 $m/s^2$ for Mars.

For the open topology, $|\vec{V}_i|$ and $|d\vec{V}_i|$ would be larger. Let's take $|\vec{V}_i|$ = 200 m/s and $|d\vec{V}_i|$ = 200 m/s, then $\frac{d\vec{V}_i}{dt}$ would be about 0.4 $m/s^2$,also one order of magnitude smaller than $\vec{g}$.

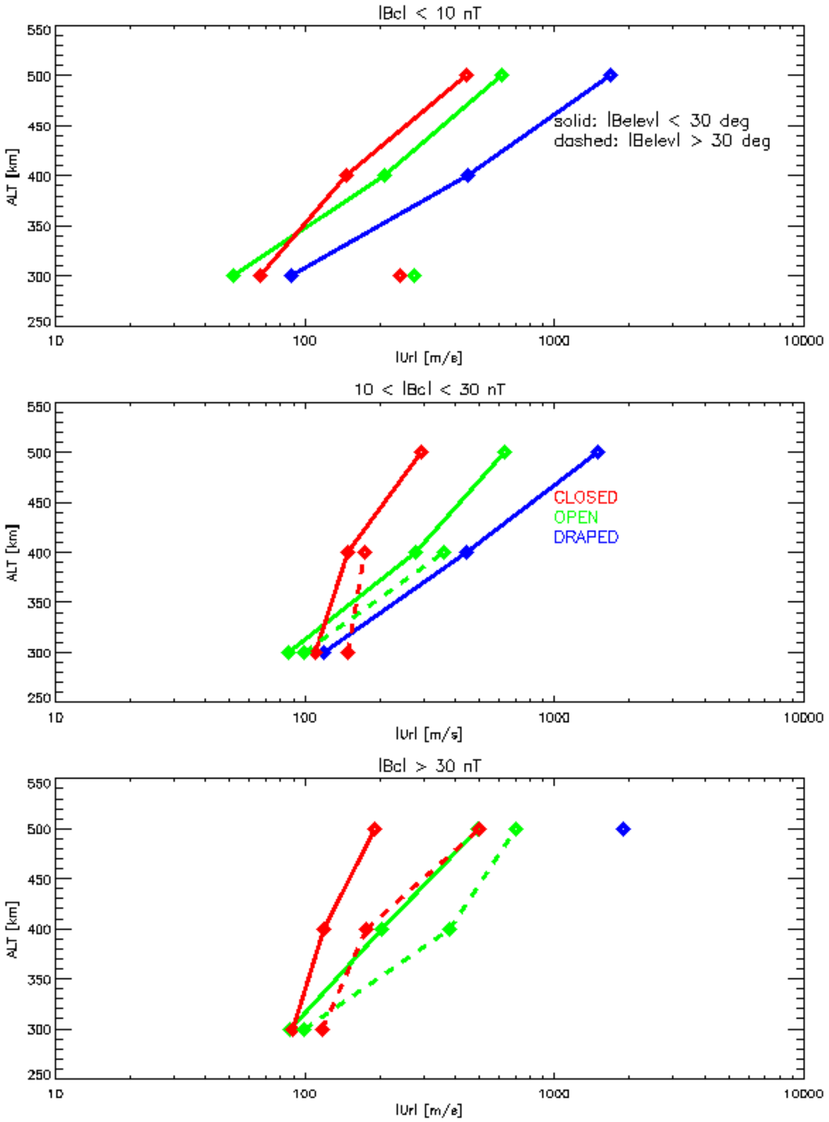


**Figure S4.** MHD simulated $O_2^+$ vertical velocity (median values) in the Martian upper ionosphere for different magnetic topology and different crustal magnetic field conditions.

## Text S4: derivation of profiles in Figure 3 and Figure 4

Here we show how the electron density and pressure profiles in Figure 3 and Figure 4 is derived. The electron density and thermal pressure ($n_e k_b T_e$) data points under low $P_{SW\perp}/P_{CMF}$ and high $P_{SW\perp}/P_{CMF}$ conditions are grouped by different altitudes with a bin width of 20 km (i.e., 190-210 km, 210-230 km, ..., 390-410 km). Each * symbol represents the median value for each data group. The dashed curves are fitted through those median values with exponential equations.

The electron thermal pressure data points for the open topology and under low $P_{SW\perp}/P_{CMF}$ are quite sparse, potentially making the results in Figure 4 less reliable.

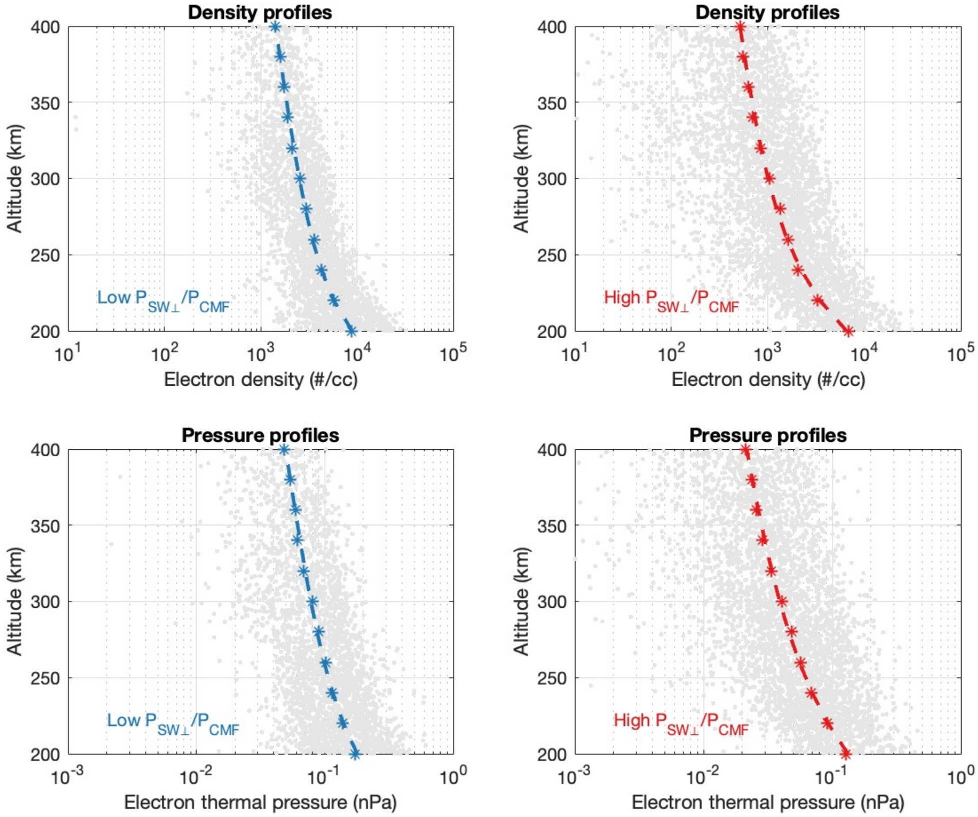


**Figure S5.** Electron density and thermal pressure profiles under low $\boldsymbol{P_{SW\perp}/P_{CMF}}$ (blue) and high $\boldsymbol{P_{SW\perp}/P_{CMF}}$ at 200-400 km for the closed topology. The grey dots represent data points. Symbol "*" represent the median values for the electron density or pressure data points within +/- 10 km (e.g., 190-210 km, 210-230 km, …, 390-410 km). The dashed curves are fitted using the median values.

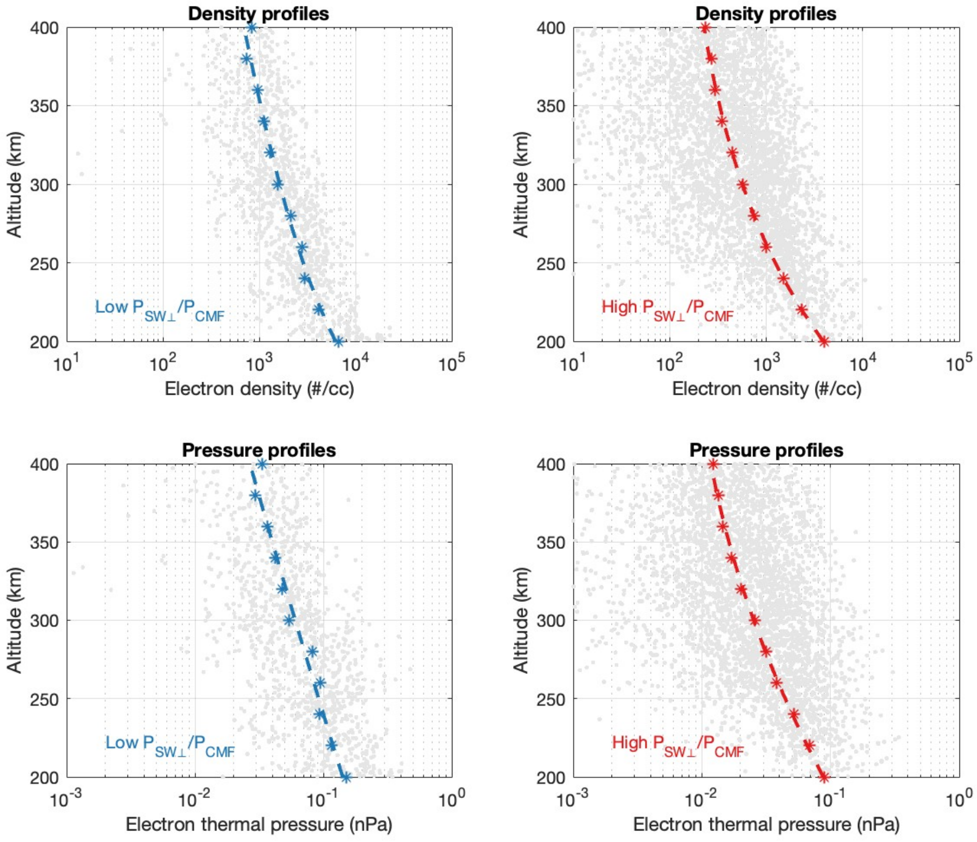


**Figure S6.** Same as Figure S5 but for the open topology.

## References

Andersson, L., Ergun, R.E., Delory, G.T., Eriksson, A., Westfall, J., Reed, H., McCauly, J., Summers, D. and Meyers, D. (2015). The Langmuir Probe and Waves (LPW) Instrument for MAVEN. Space Sci Rev 195, 173–198. https://doi.org/10.1007/s11214-015-0194-3.

Ma, Y., X. Fang, C. T. Russell, A. F. Nagy, G. Toth, J. G. Luhmann, D. A. Brain, and C. Dong (2014), Effects of crustal field rotation on the solar wind plasma interaction with Mars, Geophys. Res. Lett., 41, 6563–6569, doi:10.1002/ 2014GL060785.

Mitchell, D.L., Mazelle, C., Sauvaud, J.A., Thocaven, J.J., Rouzaud, J., Fedorov, A., Rouger, P., Toublanc, D., Taylor, E., Gordon, D. and Robinson, M. (2016). The MAVEN solar wind electron analyzer. Space Science Reviews, 200, 495-528. https://doi.org/10.1007/s11214-015-0232-1.
